# Phantom sinks and missing pollution: Legacies of a hardened number

**DOI:** 10.1073/pnas.2531787123

**Published:** 2026-01-13

**Authors:** Bruce A. Hungate

**Affiliations:** ^a^Center for Ecosystem Science and Society, Northern Arizona University, Flagstaff, AZ 86011; ^b^Department of Biological Sciences, Northern Arizona University, Flagstaff, AZ 86011

Science advances by refining estimates, but sometimes those estimates harden into infrastructure. A number, once provisional, becomes foundational. Its derivation fades, but its influence endures, shaping projections that follow and quelling the questions the estimate was meant to provoke. This essay begins with a question: What happens when estimates become canon, when models inherit numbers that look right but no longer hold? Kou-Giesbrecht et al. (1) offer a vivid answer.

**Fig. 1.**
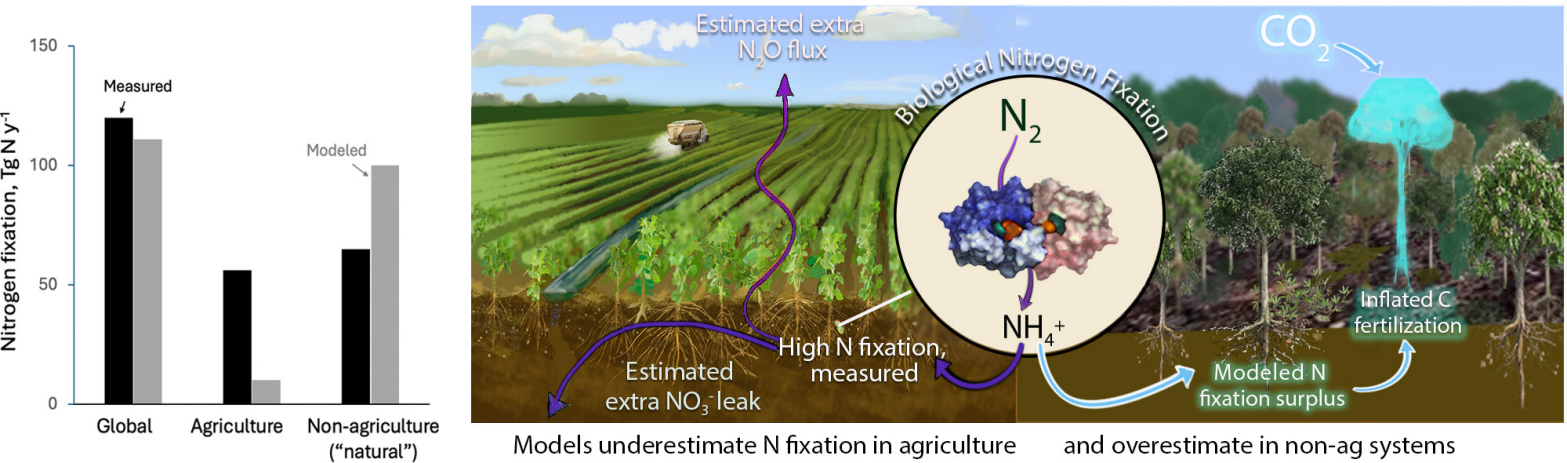
Structural misallocation of biological nitrogen fixation (bar graph on *Left*) and its consequences (image on *Right*). The bar chart compares biological nitrogen fixation (BNF; Tg N y^−1^) from empirical estimates (black bars; Ely et al., as used by ref. 1) with CMIP6 model estimates (gray bars) for global, agricultural, and nonagricultural (“natural”) ecosystems. Global totals are similar, but models underestimate fixation in agriculture and overestimate fixation in natural ecosystems, so the spatial pattern is inverted. The central lens zooms from root nodules to the molecular scale of BNF: the enzyme nitrogenase converts inert atmospheric dinitrogen (N) into ammonium (NH^+^), the reduced nitrogen used for life. On the agricultural side of the landscape (*Left*), purple arrows trace “missing” fixed nitrogen that models omit, leaking as nitrate NO_3_^−^) to waters and as nitrous oxide (N_2_O) to the atmosphere. On the natural-ecosystem side (*Right*), cyan and white arrows show surplus modeled nitrogen that relaxes nitrogen limitation, inflates CO_2_ fertilization, and creates a phantom land carbon sink.

Their reassessment of biological nitrogen fixation reveals the structural cost of relying on a 20-y-old placeholder that inflated the modeled nitrogen supply and concealed nutrient limitation of the global carbon sink (Fig. 1). This matters because biological nitrogen fixation, as represented in Earth system models, directly controls how strongly land ecosystems are projected to buffer future CO_2_ emissions. Using new, spatially explicit datasets (2), Kou-Giesbrecht et al. (1) mapped nitrogen-fixing plant abundance across natural and agricultural systems and rescaled global biological nitrogen fixation, the microbial conversion of atmospheric nitrogen (N_2_) into plant-available forms such as ammonium. Their analysis revealed a structural misallocation in nitrogen budgets: Earth System Models overestimate natural nitrogen fixation by about 35 Tg N per year and underestimate agricultural nitrogen fixation by about 46 Tg N per year (1 Tg = 1 million metric tons). The errors nearly cancel numerically, keeping the global sum intact. But that’s the problem. Once the totals add up, there’s a risk we stop asking if they line up in the first place. The error wasn’t visible in outputs. Models that look right can be wrong in the bones.

In 1999, Cleveland et al. published what became the most widely used global synthesis of biological nitrogen fixation in natural ecosystems (3). Their work improved on earlier efforts by providing biome-specific averages rather than single global means. They gathered site-level measurements, sorted by biome, and scaled up. Their methodology was cautious, but the data came from places where nitrogen-fixing organisms could be found and rates of nitrogen fixation could be measured, places where signal was likely but not necessarily representative. Cleveland and colleagues flagged the uncertainty. Their estimate was provisional, a starting point.

But once published, the estimate was repeated, absorbed, and institutionalized, influencing nitrogen cycling routines (4) and carbon sink projections (5). Models with N fixation showed ample nitrogen to fuel future CO_2_-driven carbon storage (6). Repeated often enough, what began as careful synthesis started to feel like truth.

We microbial ecologists often end papers with the plea, “put this into the models.” Cleveland and colleague’s findings were. The trouble came when we stopped asking how, because what looked like coherence was just arithmetic in disguise. The balance held together by compensating flaws: Natural systems were given too much nitrogen and agriculture too little.

Earth system models assumed enough nitrogen to sustain a robust CO_2_ fertilization effect. But when that estimate is revised, more than 10% of modeled carbon uptake vanishes because the needed nitrogen never existed: phantom nitrogen fixation supporting phantom carbon sequestration, climate optimism as artifact of inherited error. Empirical data show that forest nitrogen inputs are insufficient to sustain the modeled carbon gains (7).

Kou-Giesbrecht et al. (1) show how alignment with observations can arise from contradiction: totals that match underlain by internals that don’t.

The implications go beyond overestimated natural nitrogen fixation propping up an exaggerated carbon sink: Underestimated nitrogen fixation in agriculture in the models also obscured an uncounted nitrogen source of 46 Tg nitrogen per year, nearly four times annual nitrogen fertilizer use in the United States (8), enough to drive substantial environmental impacts (9). Some of that 46 Tg of nitrogen per year will leach into rivers (10), volatilize (11), and escape as N_2_O (12), a gas with nearly 300 times the warming power of CO_2_ [over 100 y, (13)]. As a first-order check, using conservative emission factors, the missing nitrogen could account for ~0.6 to 0.9 Tg nitrogen as N_2_O per year, about 10% of global anthropogenic N_2_O. And if a fifth of that nitrogen leaches, as is typical (14), that’s ~9 Tg nitrate-nitrogen per year—a flood of invisible pollution, unmodeled, unmeasured, unmanaged.

The phantom carbon sink and the missing pollution are consequential (15). Earth system models influence emissions targets (16), shape nutrient policy (17), and underpin adaptation plans (18). If models misallocate nitrogen, the errors move from technical glitch to distortion of consequence and responsibility.

Scaling is a way of seeing: To scale from site to biome, or from process to flux, is to decide what counts and what gets ignored. Estimation turns the invisible into consequence, which carries ethical weight: What gets scaled is taken seriously; what doesn’t, vanishes. This is a good reason to argue over scale choices. One collaborator preferred per-square-meter values, faithful to the plot. I pushed to carry numbers to hectares, the scale of management. Both are defensible. Name the leap, and why you take it.

My father taught me approximation as quantitative thinking: Round, then reckon. The answer will be close enough to think about, to see if it matters. In graduate school, a mentor reminded me of this, saying, almost offhand: “You’re allowed to multiply.” Those habits of mind make estimates and scaling up tools for confronting broader questions: If this much carbon is going into plants, how much nitrogen is needed to support it, and where might that nitrogen come from (19)? The answers are rarely definitive, but they sharpen the problems, revealing what would have to be true, making the trade-offs legible. The work of estimation is a kind of consequence-checking, a structural test for plausibility. Rough calculation is framing, not guessing. It is model-adjacent, checking models for consequence, surfacing the assumptions doing the work. It is asking: What must be true for this to hold?

Kou-Giesbrecht et al. are doing that kind of consequence-checking for nitrogen fixation and rising CO_2_. Doing so is challenging when models grow complex, and when reductionist science urges restraint, so the culture of science leans more toward caution. First-order logic feels old-fashioned, coarse, and unsanctioned, too simple for today’s models and for today’s data. But complexity makes simple checks essential. There are still things a napkin sketch can catch that a PCR reaction or a 1° grid cell never will.

Scaling in science starts with a leap, then becomes a recursive responsibility. To extrapolate is to commit to a relationship between part and whole, between rate and consequence, between what we measure and what we believe it means. Units are commitments. Estimates don’t end once numbers are published or plugged into models. They must be renewed and revisited, tested again as the system shifts around them.

This is why the most precise measurements—nitrogen concentration in g N per g tissue, excess atom fraction ^18^O in molar ratios of isotopes, or CO_2_ flux in micromoles per square meter per second—can be ecologically hollow if they’re not translated. A relative growth rate may be mathematically tidy and tell us something about organismal ecology, but until it’s anchored to land area and time, it says nothing about carbon flux, nitrogen demand, or planetary consequence. Interpreting often requires scaling. Concepts are models, but concepts without numbers are hunches. Even a rough bracket is better than an elegant sign diagram. We must ask: What would this mean if it continued? What must be true for this to be real?

When models simulate entire Earth systems, the tools we use to interrogate them must stretch too. But that doesn’t mean leaving behind the basics. Coarse estimates are useful first-order checks that can throw a flare when something is off.

Estimates remain alive as invitations, return points for the next round of questions. Kou-Giesbrecht et al. modeled that return, showing how the right question, asked again, can unearth error masked by coherence. Their work reminds us that even agreement between models and observations can conceal contradiction.

The problem wasn’t just that Cleveland and colleague’s estimate was wrong. It was that it fit. The numbers added up, or seemed to, which invited trust and may have discouraged scrutiny. The coherence was comforting but misleading. It disguised a deeper mismatch between structure and consequence. Kou-Giesbrecht et al. show what happens when that structure is interrogated.

Their results matter for more than just fixing a number. Kou-Giesbrecht et al. tested three different ways to represent biological nitrogen fixation in Earth system models: tied to evapotranspiration, to plant productivity, or also as a function of plant N and P demand. These choices matter because they affect how much nitrogen is available to plants when CO_2_ rises. When models assume high rates of nitrogen fixation in natural ecosystems, plants are less limited by nitrogen, so they respond more strongly to elevated CO_2_, causing a larger land carbon sink. But when water availability or productivity limit fixation, the CO_2_ response weakens. The result is a 40% swing in projected CO_2_ fertilization effects depending solely on how nitrogen fixation is modeled.

To narrow the range of plausible CO_2_ fertilization responses in Earth system models, Kou-Giesbrecht et al. used an emergent constraint, “emergent” because it arises not from any single model or external measurement alone, but from a systematic relationship that emerges when comparing multiple model outputs to one another and to observations. It works by tracing a line: Models that overestimate nitrogen fixation tend to overestimate the land carbon sink. By comparing model spread to real-world measurements, Kou-Giesbrecht et al. shrink the range of plausible future carbon trajectories. This correction to the global rate and distribution of biological nitrogen on land exposes the cost of the inertia that preceded it.

Quantitative estimates are communicators, like words. Used too often without reexamination, both lose their tether to the circumstances that gave them meaning. Estimates become outdated; words become cliché. The remedy is recursion: Estimate, draft, scale, edit, reassess—because if we don’t, the infrastructure calcifies.

Kou-Giesbrecht et al. (1) show how alignment with observations can arise from contradiction: totals that match underlain by internals that don’t. Models that match data create trust, but that trust should rest on structure, transparency, and humility, not just on globally aggregated sums. Agreement should be earned, not reverse-engineered.

This essay began with a question: What happens when approximations become canon? Kou-Giesbrecht gave one answer. Here’s a corollary: When estimation remains recursive, transparent, and tied to consequence, it becomes care, the kind of care that keeps our models honest and our science from drifting too far from the world it claims to describe.
